# Sensitive and rapid detection of cholera toxin subunit B using magnetic frequency mixing detection

**DOI:** 10.1371/journal.pone.0219356

**Published:** 2019-07-05

**Authors:** Stefan Achtsnicht, Christian Neuendorf, Tobias Faßbender, Greta Nölke, Andreas Offenhäusser, Hans-Joachim Krause, Florian Schröper

**Affiliations:** 1 Institute of Complex Systems, Bioelectronics (ICS-8), Forschungszentrum Jülich, Jülich, Germany; 2 RWTH Aachen University, Aachen, Germany; 3 Fraunhofer Institute for Molecular Biology and Applied Ecology IME, Aachen, Germany; The Ohio State University, UNITED STATES

## Abstract

Cholera is a life-threatening disease caused by the cholera toxin (CT) as produced by some *Vibrio cholerae* serogroups. In this research we present a method which directly detects the toxin’s B subunit (CTB) in drinking water. For this purpose we performed a magnetic sandwich immunoassay inside a 3D immunofiltration column. We used two different commercially available antibodies to capture CTB and for binding to superparamagnetic beads. ELISA experiments were performed to select the antibody combination. The beads act as labels for the magnetic frequency mixing detection technique. We show that the limit of detection depends on the type of magnetic beads. A nonlinear Hill curve was fitted to the calibration measurements by means of a custom-written python software. We achieved a sensitive and rapid detection of CTB within a broad concentration range from 0.2 ng/ml to more than 700 ng/ml.

## Introduction

Cholera is a waterborne disease [1–2] which leads to a life-threatening acute watery diarrhea [2–5]. In 2017 over 1.2 million cases of cholera resulting in over 5600 deaths have been reported to the World Health Organization (WHO) [3]. It is estimated that only 5–10% of the cases and deaths are reported, which leads to the estimation (from 2014) that there are between 1.3 and 4 million cases each year, leading to some 21 to 143 thousand deaths/year worldwide [6]. The number of reported cases and deaths in 2017 was much higher than at the time the total numbers were estimated (2014), due to an outbreak in Yemen which was responsible for 84% of the WHO’s reported cases and 41% of the deaths alone [3]. As cholera is mainly affecting the poor [7] and is often spread in suburban areas, it is important to have a cheap on-site test which is not depending on the availability of well-equipped laboratories. Cholera has a short median incubation period of 1.4 days, and the symptoms are visible within 4.4 days (95%) [4]. As it can become epidemic, quick tests are needed. The WHO’s global task force on cholera control named the development and the availability of such a rapid test as part of one of their 3 axes to fight cholera [7].

Cholera is a result of intoxication with *Vibrio cholerae (V*. *cholerae)*, a bacterium which produces cholera toxin (CT). Around 200 serogroups of *V*. *cholerae* are known today, two of which (O1 and 0139) are the most dangerous and can become epidemic [1–2,4]. CT is a heterohexameric toxin, consisting of a single A subunit (CTA) and a homopentameric B subunit (CTB) [8]. With this structure, it belongs to the group of AB_5_ toxins, which are medically important toxins that constitute important virulence factors [9–10]. The A subunit of CT is the catalytic active part which leads to the disruption of essential host functions. It can be divided into two sections [2,9]. The first section (CTA1) is responsible for the toxicity by means of an increased generation of cyclic adenosine monophosphate (cAMP) in the cytosol which leads to a chloride ion (Cl-) secretion [2,11]. To keep the osmolality, this results in a water outflow into the intestinal lumen and cell death. This increased water outflow leads to the symptomatic diarrhea and therefore to a reduction of the blood volume [8]. The second section CTA2 is responsible for the noncovalent anchoring of CTA inside the homopentameric CTB. The ring-shaped CTB binds to the monosialotetrahexosylganglioside (GM1) receptors on mammalian intestinal epithelial cells with high affinity. As each monomer of the CTB pentamer has a receptor binding site, it can bind to five GM1 receptors at a time. It has been shown that only one functional binding site is sufficient for the intoxication pathway, but with a reduced activity [11]. The whole CT is then endocytosed into the cell and retrograde transported to the endoplasmic reticulum where CTA1 is separated [9,11,12]. CTB is not toxic, and CTA needs CTB as a transport vehicle inside the cell. Because of this, only the whole CT complex is intoxicating cells, and not one subunit alone.

The detection of CTB is not only relevant for the diagnosis of cholera or drinking water safety analysis but it is currently also used in different other applications. For example, it is used as a vaccine adjuvant or anti-inflammatory agent [2] or as a mucosal immunomodulatory agent [13].

Different techniques exist for the detection of Cholera or CTB, respectively [14–18]. One form is the direct cultivation of *V*. *cholerae* on a medium which is considered as the gold standard [14]. This method has the advantage that the detection sensitivity can be increased by longer incubation, but that is time consuming (about 24 hours [14,15,17,19,20]), and the amount of health-threatening material is amplified. Additionally, special microbiological equipment and incubators are required, as well as microbiologically skilled personnel. With this approach, only living bacteria can be detected. Different rapid diagnostic tests based on immunochromatographic methods are commercially available and have been compared to the gold standard during different outbreaks of cholera [20]. Another possibility is polymerase chain reaction (PCR) to detect the bacteria’s deoxyribonucleic acid (DNA) [15]. Here it is important that the DNA is not destroyed during sample handling or due to previous environmental conditions. As expensive laboratory equipment and skilled personnel is required, it is not so practicable for in-field use. With this technique, it is not possible to detect the toxin but only the bacterial DNA. As the toxin itself leads to the symptoms, it might be preferable to detect it directly, especially since the efficiency of antibody-based assays recognizing *V*. *cholerae* in general are highly dependent on the binding specificity between antibody and antigen. Thus immune-based approaches, which can specifically detect the strains harmful for humans from all different serotypes of *V*. *cholerae*, are difficult to establish. As toxins can withstand different environmental conditions than the bacteria and are not affected by antibiotic treatment, this direct testing can be favorable. It should also be mentioned that, for example, in cases of bioterrorism, where the pure toxin is used instead of the whole bacteria, a bacteria/DNA based detection method would not work. The authors in [18] mention that there is an “urgent demand for rapid and accurate determination of bacterial toxins” and give CT as an example. For the direct detection of the toxin, the nontoxic B subunit can be used. As mentioned before, the A subunit alone is not able to intoxicate a patient. Therefore the CTB detection is sufficient. As CTB has a homopentameric structure which binds up to five receptors at a time, it is possible to bring more than one marker to a CTB unit at a time. This possibility of more than one binding site is used, for example, in the latex agglomeration test [15], in colorimetric assays or in dynamic light scattering assays [21]. Another approach is to use fluorescence labels or radioimmunoassays [22]. An often used method is the enzyme-linked immunosorbent assay (ELISA) [15,22]. Superparamagnetic beads (MBs) have been used to capture CTB magnetically and to afterwards measure them with an attached fluorescent dye or electrochemical marker [23]. Here the MBs are used as handles, like in separation or targeting setups [24].

In this research, we decided to directly use superparamagnetic beads as a marker to determine the achievable detection limit and the quality of the calibration curve. In modern bioanalytical and biomedical applications [16,24,25], MBs are widely used as labels, handles, or both. Different labels can be used in immunologic detection technologies, for instance, enzymes, fluorophores, radioisotopes, or magnetic beads [26,27]. In ELISAs, the linked enzymes catalyze a reaction leading to a color change. This behavior as well as the reaction of the fluorophores might be difficult to detect if the sample is colored or turbid. Another disadvantage of markers like radioisotopes or fluorophores is that their signal changes over time because they have a half-life or undergo photo bleaching. In contrast, the signal of the MBs do not change over time and, therefore, can be read out at a later time or read out later again [28]. For the detection of MBs, different magnetic sensors can be used, for example, coils, giant magnetoresistance (GMR) sensors [29], and superconducting quantum interference devices (SQUIDs) [30]. Common techniques for magnetic measurements are, for example, relaxometry [31], susceptometry [32], and nuclear magnetic resonance (NMR) [33]. We used the magnetic frequency mixing technique [34–38] to develop a simple rapid detection system for CTB. In [36–38], this technique has been compared with ELISA measurements and its applicability for immunoassays has been shown. As CTB is in many application cases solved in a liquid-like medium (drinking water, diarrhea stool), a detection method which can directly make use of this is favorable. Therefore we used a sandwich immunoassay inside a three-dimensional (3D) immunofiltration column.

## Materials and methods

### Magnetic sandwich immunoassay

**Fig 1:**
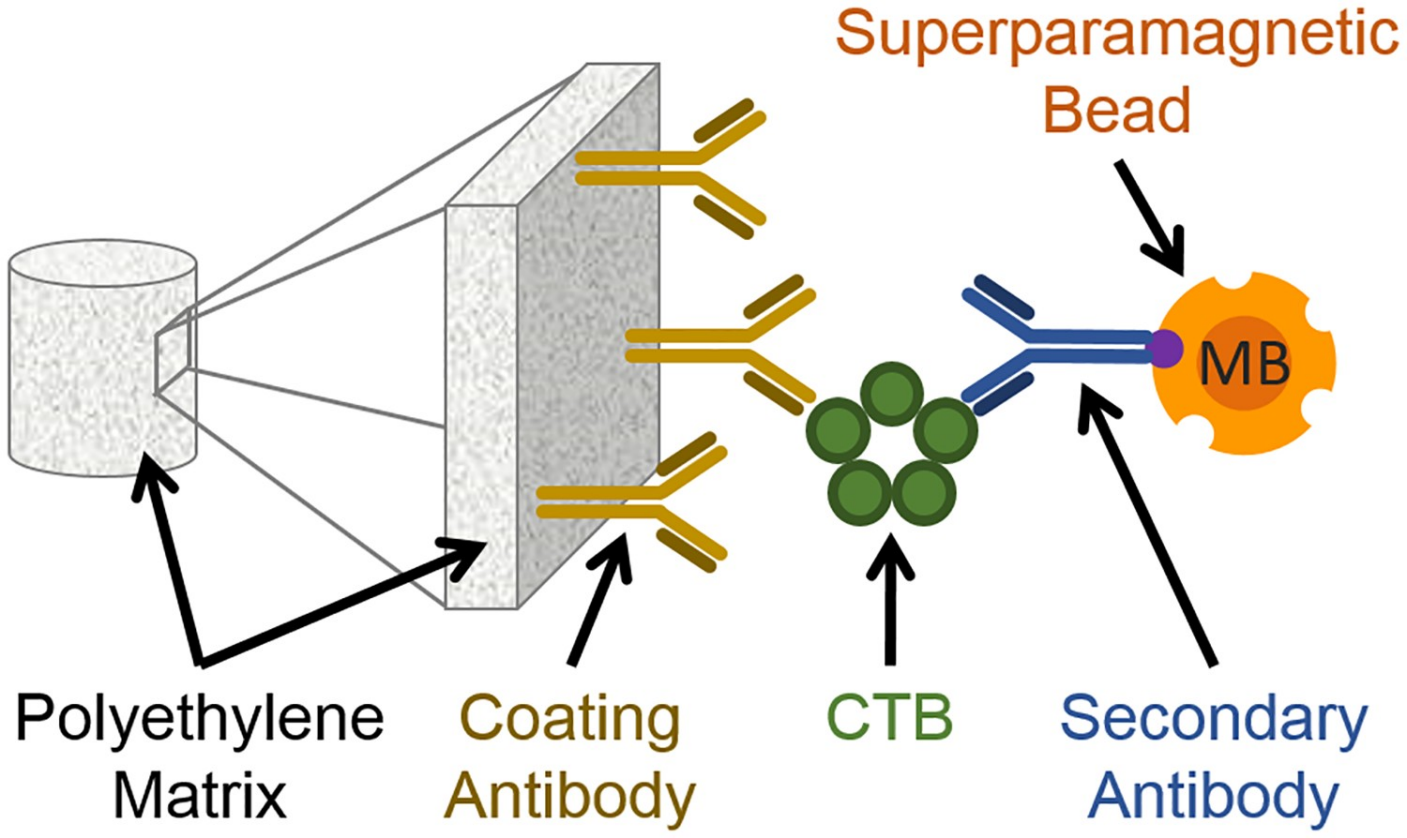
Schematic representation of the magnetic sandwich immunofiltration assay. A coating antibody (also primary antibody, brown) is immobilized on a porous polyethylene matrix. The Cholera toxin’s subunit B (CTB, green) is captured by the antibody when the CTB-containing solution is flushed over in gravity flow. As the next step, the biotinylated secondary antibody (blue) is flushed over, which also binds to the CTB. Finally the streptavidin-coated superparamagnetic beads (MB, orange) bind to the biotin moiety of the secondary antibody.

In this work, we use a sandwich immunoassay approach [18] to qualitatively and quantitatively measure CTB inside a tap water sample. The specific composition of the tap water as used from the regional water supplier is given in S1 Table. A schematic representation is shown in Fig 1. Our target CTB dissolved in tap water is first captured by an immobilized antibody and afterwards labeled with a second biotinylated antibody which is connected to a streptavidin-coated MB. These beads act as labels for the magnetic frequency mixing detection described below. In this work, we use two different types of beads from the company micromod Partikeltechnologie GmbH (Rostock, Germany), called A (hydrodynamic diameter 75 nm) and B (hydrodynamic diameter 1010 nm), as described in the following.

Monoclonal antibodies (mAb) IgG2a mouse anti-cholera subunit B toxin specific mAb E6 and mAb E10 for coating immunofiltration columns and magnetic beads, respectively, were purchased from HyTest Ltd, 20520 Turku, Finland. They are sold as Cat.# 2C4 MAb E6—Lot:15/09-C4-E6, concentration = 4,6 mg/mL in PBS, pH 7,4 (+0,09% sodium azide) and Cat.# 2C4 MAb E10—Lot:15/10-C4-E10, concentration = 9 mg/mL in PBS, pH 7,4 (+0,09% sodium azide), respectively. According to their datasheet [39], the antibodies are generated by hybridoma clones, which have been derived from hybridization of Sp2/0 myeloma cells with spleen cells of Balb/c mice immunized with purified CTB. They were produced for use in ELISAs and stored until usage, as recommended by the manufacturer [39]. To bind mAb E10 to the streptavidin coated magnetic beads, the antibodies were biotinylated using commercially available EZ-Link NHS-PEG_4_-Biotin (Thermo Fisher Scientific, Erlangen, Germany) following the manufacturer’s recommendations [40]. This combination is a result of our preliminary ELISA measurements that we performed to find out if it is better to use the same antibody twice (for binding to the column and labeling with the MBs), or if we get better results when using two different antibodies for capturing and labeling, and which antibody works better in which role. The results of these preliminary experiments are shown in S1 Appendix, S1 Fig and S2 Table. We chose mAb E6 for coating and mAb E10 as secondary antibody because this combination yielded the highest measuring signals, a broad detection range and lowest detection limits. Subsequently, this antibody combination was transferred to the immunomagnetic assay. The change from a buffered system to the direct use of tap water was also tested. We verified that antibody binding properties are not negatively affected in tap water and thus found no significant differences in the magnetic immunoassay. From former measurements as well as from direct communication with the manufacturer, it is known that mAb E6 binds to CTB with cross reactivity to the heat-labile enterotoxin from Escherichia coli (EtxB) with almost the same affinity. In contrast, mAb E10 doesn’t show this behavior. By using mAb E6 for coating and mAb E10 as secondary antibody we might still bind the cross reacting EtxB in the column, but it will not be labeled and therefore not be detected. Additionally, by using antibodies which bind to two different non-overlapping epitopes, also the monomer of CTB should be detectable.

The streptavidin-coated superparamagnetic beads used in this research were custom-made by micromod Partikeltechnologie GmbH (Rostock, Germany). Two different types of particles were used. The first bead called A (Article-No. 104-19-701, Lot 04117104) in this work has a hydrodynamic diameter of about 75 nm (Z-Average, Polydispersity index (PDI): 0.12). The biotin-binding capacity is 434 pmol, and they have an iron content of 6 mg/ml. Type B (Article-No. 05-19-502, Lot 0471805) has a bigger hydrodynamic diameter of about 1010 nm (Z-Average, PDI: 0.227). The biotin binding capacity is 150 pmol, and it also has an iron content of 6 mg/ml. All given data are according to the manufacturer.

The recombinant cholera toxin B subunit (rCTB) was expressed using the *Escherichia coli* strain BL21 (fhuA2 [lon] ompT gal (λ DE3) [dcm] ΔhsdS λ DE3 = λ sBamHIo ΔEcoRI-B int::(lacI::PlacUV5::T7 gene1) i21 Δnin5), [41]. It was carrying a pET-22b(+) plasmid (pET-22b(+) DNA–Novagen, Merk KGaA, Darmstadt, Germany) with an artificially CTB gene. It has the same protein primary structure as the protein data bank entry 3CHB_D [42]. It was shown that the produced rCTB behaves similarly in an ELISA as commercially available CTB (C9903-.5MG, Sigma-Aldrich Co. LLC., St. Louis, Missouri, USA).

The sandwich immunoassay was performed using commercially available ABICAP columns (abicap HP columns, hydrophobic) from Senova Gesellschaft für Biowissenschaften (Senova, Weimar, Germany).

### Preparation of the columns

The filters inside the columns were hydrophobically equilibrated. They were placed inside a beaker with ethanol using a desiccator with an applied under pressure of -0.8 bar to remove air from the matrix pores, followed by washing steps. Details of this general procedure are given in [38].

Afterwards, the filters were coated with mAb E6 antibodies by flushing the column with 500 μL mAb E6 in carbonate buffer (c = 20 μg/ml) in gravity flow, followed by 30 minutes incubation time. The flow-through was collected and afterwards reapplied over the respective column, followed by another 30 minutes of incubation time.

To saturate uncoated parts of the matrix, two times 500 μl bovine serum albumin solution (BSA, 10 mg/ml in phosphate-buffered saline (PBS), pH 7.4) were flushed through by gravity in 20 minutes each, followed by a washing step with 750 μl PBS. For storage, the columns were closed at the bottom, filled with PBS, closed at the top, and placed in a refrigerator at 4°C. In this work, we used storage times of a few days.

### Performing the assay

First the antigen-containing solutions were prepared. The rCTB was solved in local tap water (Aachen, Germany, composition see S1 Table). Concentrations of 0, 10, 25, 50, 75, 100, 250, 500, 750 ng/ml were used. For both bead types, each sample inside the calibration set was prepared on two different columns in order to take the biological fluctuations between measurements into account. 500 μl of the respective rCTB containing solution were flushed over the columns by gravity flow and incubated for 60 minutes at room temperature. To remove unbound rCTB, a washing step with 750 μl PBS was done.

10 μl PBS solution with biotinylated mAb E10 (mAb E10bio) (10 μg/ml) were flushed over and incubated for 30 minutes. Afterwards, two washing steps with 750 μl PBS with Tween (PBS-T, 0.1%) were performed.

The magnetic bead-containing solution (500 μl, 180 μg/ml of MB type A or B) was flushed over by gravity flow, followed by 30 minutes of incubation time. To remove unbound magnetic beads, which would lead to a false positive signal, two washing steps with 750 μl each were performed, one with PBS-T and the other with PBS solution.

### Magnetic frequency mixing detection

**Fig 2:**
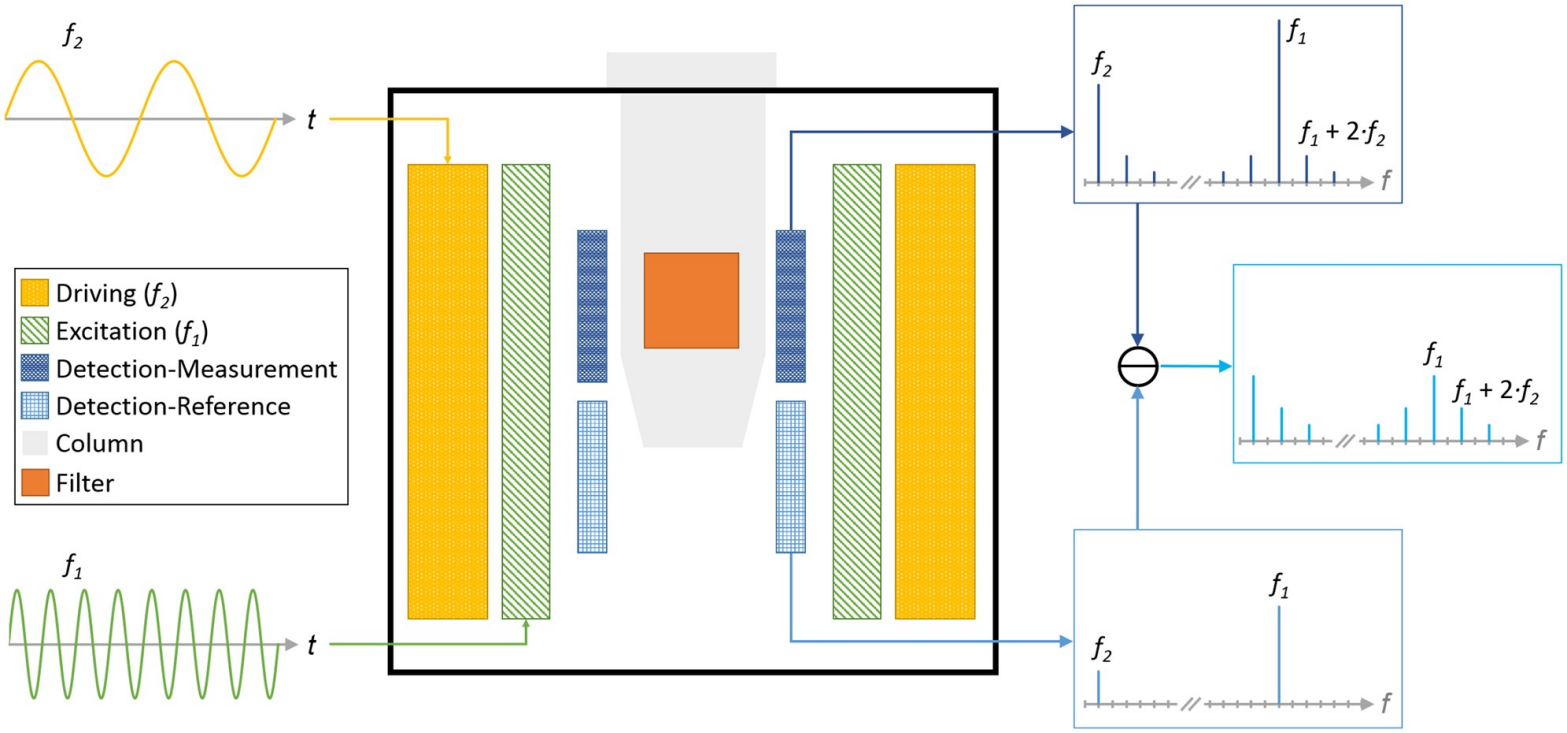
Schematic drawing of an axial cut through the measurement head with schematic view of the working principle. The measurement head consists of two coils for providing the low driving (*f*_*2*_, yellow, dotted) and high excitation (*f*_*1*_, green, diagonal lines) frequency magnetic field. The differential detection coil is shown with its two compartments for measurement (fine dark blue grid, top) and reference (light blue grid, bottom). The head is designed so that the sample filter (orange) inside the column (gray) is resting at the most sensitive position of the measurement part of the detection coil. Therefore the measurement-coil (dark blue) picks up the MBs signal, as well as, the direct induced signal. The reference-coil (light blue) picks up only the direct induced signal. As they are connected in a differential configuration the resulting signal does not contain these direct induced signals anymore.

To detect the superparamagnetic beads, we used the magnetic frequency mixing technique [34]. The sample is placed inside a measurement head which consists of three coils. The basic working principle of the measurement head is sketched in Fig 2. The outermost coil is used to apply a magnetic driving field *f*_*2*_ with a frequency of about 63 Hz. It has a maximum amplitude of a few mT, which drives the superparamagnetic beads in the nonlinear crossover towards saturation at the extrema of the driving field. The next coil is generating the magnetic excitation field *f*_*1*_ with a frequency of about 40.545 kHz. This field is used to probe the magnetization state of the beads. When the beads are already saturated by the driving field, the beads’ response to this excitation field is weak, whereas at driving fields close to zero, the excitation field generates a magnetization and therefore a strong response. Because of this interaction, the response amplitude to this excitation field oscillates with a frequency of 2·*f*_*2*_. All beads used in this research are superparamagnetic, which means they have a non-linear magnetization curve without a remnant magnetization at zero applied field (no hysteresis) [24]. This behavior together with the two oscillating fields leads to the generation of new frequencies which follow the scheme: *f*_*new*_ = *m*·*f*_1_ ± *n*·*f*_2_, where *m* and *n* are integer numbers. Without a static magnetic offset field, the mixing component *f*_*new*_ = *f*_1_ ± 2·*f*_2_ shows the highest signal and is typically used for detection of the beads. The MBs and the excitation fields induce a signal in the detection coil (innermost coil). This detection coil is made of two sections connected in series (called measurement and reference coil) in a differential configuration. Both sections have the same characteristics, like number of windings and diameter, but are wound in opposite directions. Due to this differential coil setup, the very strong directly induced signal of the excitation coils is cancelled out, whereas the sample with the bound MBs is only placed inside the measurement section and is therefore not cancelled out. This induced voltage is then amplified and filtered, and the amplitudes of the mixing terms can be determined. For example, in [36,38,43] this is done in a two-stage multiplication process, whereas in this work, we used direct digitalization of the signal trace and Fast Fourier Transform (FFT) analysis. This was chosen because we used different beads in this research, and different beads might create different phase shifts between the excitation and the detection signal traces, as shown in [44]. With the two-stage multiplication process, it might then be needed to change the phases of the reference signals with which the measured signal is multiplied during the demodulation, according to each bead type. By using the FFT, we do not need to multiply our signal with some reference signals, and therefore, no changes are needed when changing to a bead type which might have a different phase behavior.

### Measurement and analysis

The columns prepared according to the above protocol were placed inside the measurement head of the digital magnetic reader (photograph see S2 Fig). The reader was operated in continuous measurement mode, taking 1 million samples with a sampling rate of 1 MegaSample per second, followed by a break of about 2 seconds between the measurements. This results in an FFT frequency resolution of 1 Hz. With the help of a light barrier mounted inside the measurement head, the time point at which a sample column is entered was easily detectable. The column was left inside the reader to reach equilibrium temperature and stable measurement values at the mixing frequency *f*_*new*_ = *f*_1_−2·*f*_2_.

For each bead type and each rCTB concentration, the mean value and standard deviation were calculated and loaded into our python software to find the optimal fit parameters and to determine the limit of detection.

### Calibration

To obtain information about the sample, we need a calibration model to advance from the measured values via the analog to digital converter (ADC) to the concentrations of the analyte CTB. To construct a calibration set, we used the previously prepared solutions with different known concentrations of CTB in tap water and washed them over the filter. To account for imperfections like less than 100% binding efficiency, a broad range of concentrations is used, and the MBs are applied in excess. For fitting of the calibration data, we used the Hill function in the form

$$y=end\cdot \frac{x^{n}}{k^{n}+x^{n}}.$$ (1)

Here, *x* equals to the concentration of CTB and *y* to the measured signal. So just the two parameters *k* and *n* need to be fitted. This function can be used if the blank value is subtracted from the measured signals.

The Hill function can be inverted (see S2 Appendix) to obtain

$$x=k\cdot {\left(\frac{y}{end-y}\right)}^{\frac{1}{n}}.$$ (2)

The inverse can be used as long as the measured value *y* is smaller than the value determined for the parameter *end* during the fitting procedure.

## Results

### Calibration software and prediction method

**Fig 3:**
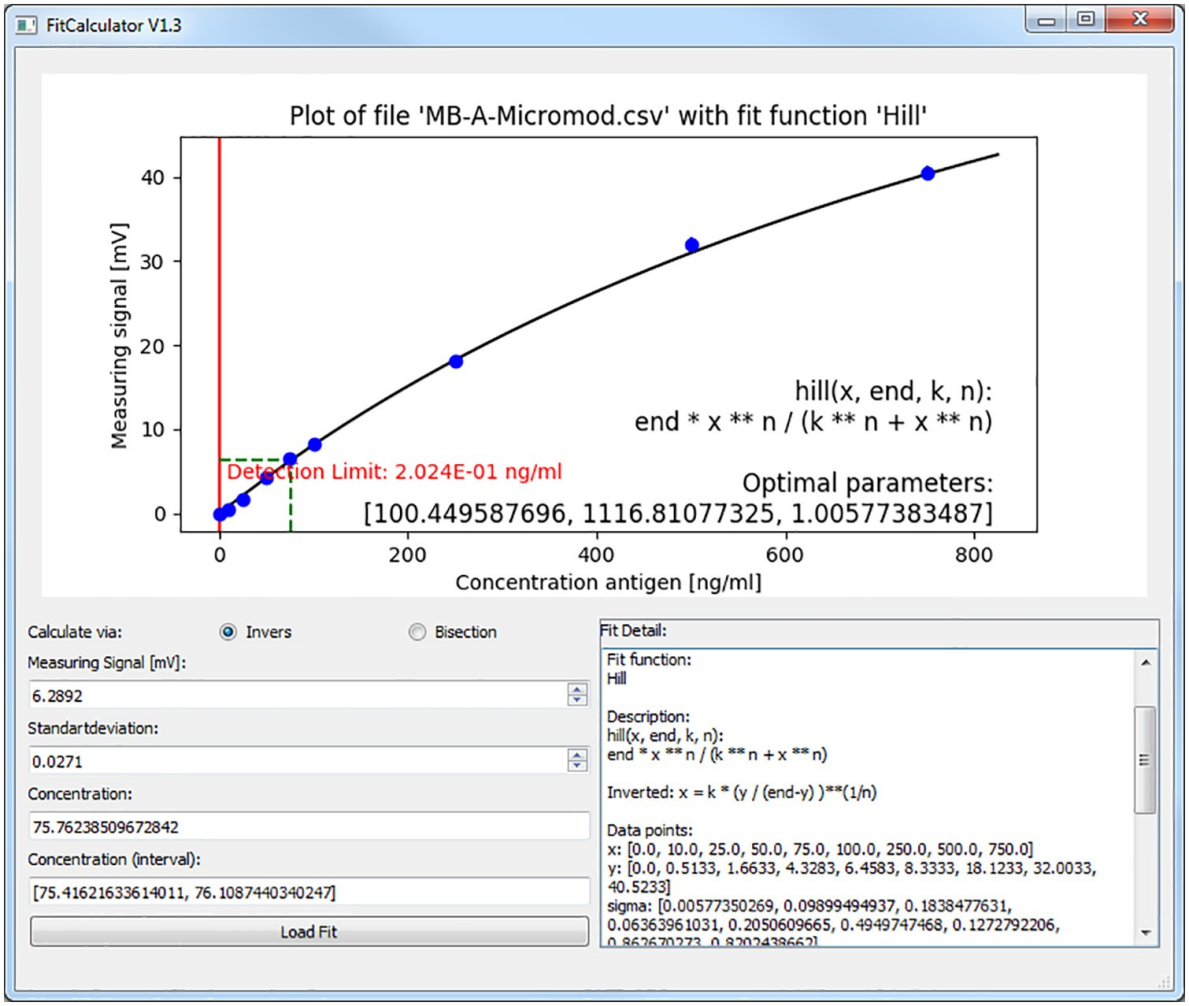
Screenshot of the software to determine the concentration of an unknown sample. The previously saved fit result for magnetic bead A has been loaded and its calibration points as well as their standard deviation (blue) and the detection limit (red) is displayed. A sample with a spiked concentration of 75 ng/ml was analyzed. A mean value of 6.29 mV with a standard deviation of 0.03 mV was measured and entered. This point is indicated on the calibration curve (black) by green lines and the range is graphically shown with the help of purple lines. For the given values, a concentration of 75.8 ng/ml was found. Together with the standard deviation, this results in a concentration in the range between 75.4 and 76.1 ng/ml.

For easy determination of the fit parameters as well as for calculating the target concentration inside an unknown sample, we wrote software in python 3 with a graphical user interface (GUI) (see Fig 3 and S3 Appendix). We checked the repeatability of the magnetic measurement by performing several subsequent measurements (n = 10) while the sample is inserted in the measurement head, and found that the statistical error of these individual measurements is in the range of a few per mil for larger signals. For example, the relative standard deviation of the 75 ng/ml spiked sample was 0.5%, see Fig 3. For very small signals, the voltage noise of the electronics becomes increasingly important. We also did repetitive magnetic measurements by removing and re-entering the columns in the measurement head, in order to check if sample-positioning inaccuracies influence our measurement results. These repetitions also yielded statistical errors in the same range as the repetitive measurements without removing the sample. The whole assaying process was repeated by performing biological duplicates for the spiked samples and triplicates for the blank reference. The latter one turned out to be the dominant source of statistical variations. Hence, we chose to take their statistical variation for our error bars. All statistical variations are low enough to yield reliable results. From these individual measurements we calculated the mean and standard deviation. The limit of detection (LOD) is determined by calculating

$${Detectionlimit}_{ADC}=Blankvalue+3\cdot {Standarddeviation}_{Blankvalue}.$$ (3)

If the *Blankvalue* has already been subtracted, the detection limit becomes simply three times the standard deviation of the blank measurements. The corresponding minimum detectable concentration is calculated by plugging *Detectionlimit*_*ADC*_ into the inverse Hill function (see S2 Appendix). This is a common definition of the LOD [21–23].

### Results of the assay and calibration

For both magnetic bead types used in this work, calibration curves with CTB concentrations between 0 and 750 ng/ml were recorded. For each bead type and each concentration of CTB, two columns were prepared, except for the blank value (no CTB in the sample) which was done three times because its standard deviation mainly determines the LOD and therefore its standard deviation should not be underestimated.

**Fig 4:**
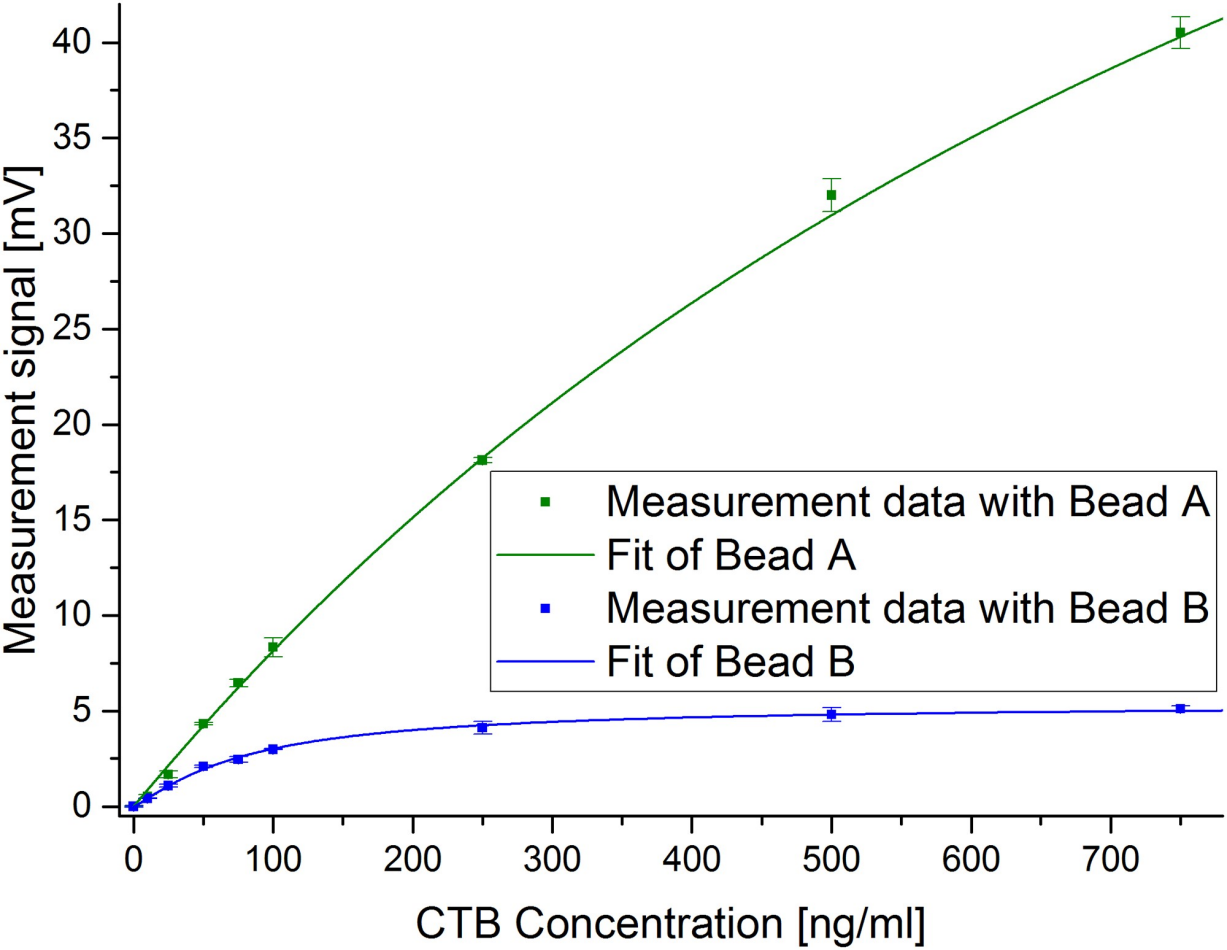
Measured magnetic signals of samples with different CTB concentrations. The mean values and the corresponding standard deviations are depicted as a function of CTB concentration for both types of magnetic beads. The Hill fit curves are also shown.

For fitting, we used the mean value and the standard deviation for each concentration. The fit performed with bead type A is given in Fig 3. In Fig 4, both fit curves are shown together. The measured values for CTB showed the characteristic behavior of a signal that increases with concentration and crosses over into saturation at higher concentrations. This saturation can have biological reasons. For instance, all binding sites in the filter are occupied or blocked by neighboring bound beads.

For type A, we measured signals ranging up to over 40 mV after subtraction of the blank value. A detection limit of 0.2 ng/ml was found. The bigger bead type B showed signals of more than 5 mV after subtraction of the blank value. With this bead type, a limit of detection of 3.1 ng/ml was found. The coefficient of determination (R^2^) was 0.999 and 0.997, respectively. The Hill curve obtained with bead B looks much flatter and more saturated, in contrast to bead A. In addition, the standard deviation is more pronounced, especially for higher concentrations.

## Discussion

With both bead types, it was possible to perform magnetic sandwich immunoassays to measure the concentration of CTB inside spiked tap water samples. With the software for fitting, we were able to find the parameters of the Hill function and determine the limit of detection in both cases.

By comparing the trace of the fit functions (Fig 4), we can see that the columns with bead type A gave higher signals in comparison to the ones using bead B. Furthermore, it is obvious that for type B, the signal goes into saturation, while for A this is not the case, even though it produces an almost 8 times higher signal at the highest concentration (750 ng/ml CTB). Because of this, an electronic saturation can be ruled out. It might be that due to the bigger size of bead B, more than one secondary antibody is binding to the same MB, or that there is sterical hindrance due to the MB’s size, preventing other MBs to bind to free secondary antibodies. For the more than tenfold smaller beads of type A, these effects are less likely. As the secondary antibody binds to one of the monomers of the pentameric CTB, more than just one MB might be able to bind to one target inside the filter. This would lead to an increased signal, whereas for the bigger bead B, this is more unlikely to happen due to steric hindrance.

With our python software, an easy-to-use solution has been created for nonlinear fitting (in this case using the Hill function). If mean and standard deviation have been determined for each calibration point, the user can decide if the standard deviation should be taken into account during fitting. The use of the standard deviation leads to better and more reliable fit results, as measurements with higher deviation are weighted less. By using a nonlinear fitting, it is possible to quantify the target concentration over a much wider range of target concentration, in contrast to often used linear fitting. If we applied linear fitting for our measurement with bead B (Fig 4), a good fit would only be possible for concentrations up to about 100 ng/ml. Our nonlinear Hill fit, however, allows to extend the range to over 700 ng/ml with excellent fitting quality. By using the inverse Hill function, it becomes possible to determine the concentration of an unknown sample, for example, on a microcontroller or on a PC using the presented software.

Bead B shows a low detection limit of 3.1 ng/ml for CTB. This detection limit was even lowered by using beads of type A. Here a very low detection limit of only 0.2 ng/ml was found. It can be seen very well in these experiments that the type of bead plays an important role for the detection limit of a magnetic assay. By changing the bead type, the LOD could be lowered by more than an order of magnitude without changing anything else in this sandwich immunoassay.

For a tap water sample with a spiked CTB concentration of 75 ng/ml using bead A, a measurement value of 6.29 mV with a standard deviation of 0.03 mV was measured (compare Fig 3). This leads to a concentration of 75.8 ng/ml (Interval from 75.4 to 76.1 ng/ml), corresponding to a percent recovery of 101.1%.

Dragunsky et al. found an LD_50_ for intraperitoneally (i.p.) applied CT of 33.3 μg ± 7.3 μg for swiss male mice (12–14 g) [45]. Gill’s review about lethal amounts of bacterial toxins lists an LD_50_ auf 250 μg/kg for mice [46]. In the review paper by Levine et al., it is mentioned that in human experiments with CT, typical cholera symptoms cannot be seen at doses of 2.5 μg, but are visible at 5 μg, and strong at 25 μg CT [47].

While cultivation methods are very sensitive, they take a relatively long time of about 24 hours [14–15,17,19–20]. In [18], different immunoassays for the detection of CT or CTB are reviewed, for example, using the induced aggregation of gold nanoparticles with an detection limit of 3 μg/ml CTB, or an impedimetric immunoassay (1 ng/ml CTB). By using electrophoretic collection of the toxin, magnetically supported binding of MBs to the bound toxins and optical readout, a detection limit of 0.1 pg/ml CT was found, using a factor of 2.5 for calculating the LOD instead of the usual factor of 3 [48]. Another approach they report is using an electrochemical immunosensor with potassium ferrocyanide molecules as markers and liposomic magnification, leading to 10^−15^ g/ml LOD for CT [49]. In this study, the authors mention that organic solvents have been used which are suspected to be carcinogenic. In [22], different detection methods for CTB are compared together with their assay times. LODs of 0.34 ng/ml were found for dually tagged liposomes, 0.49 ng/ml for ELISA, and 6.19 ng/ml for fluorescein-labeled antibodies, with average assay times of about four hours. With an enzymatic radioimmunoassay, a detection limit of 0.1 ng CT/ml and 16 h assay time have been reported. In [50], an ELISA is presented which is able to detect CT consistently at 25 pg/ml, with a total incubation time of 3 hours. In that test, it was found that in over 50% of the stool samples, the CT concentration was lower than 1 ng/ml. With fluorescence and electrochemical microfluidic biosensors, the LODs for CTB were 6.6 ng/ml and 1.0 ng/ml, respectively [23]. These methods have an assay time of one hour, which is reported to be faster than GM1-ELISA (5 hours) and latex agglutination tests (20 hours). For the reversed passive latex agglutination VET-RPLA kit (Oxoid Limited, Hampshire, England), a detection limit of 1–2 ng/ml is reported, but 20–24 hours of incubation time are needed between sample preparation and readout [15]. Starting with precoated columns from storage, the total time for performing the assay is 128 minutes in our case, with a limit of detection of 0.2 ng/ml for bead A. The 120 minutes of incubation take most of that time. This incubation time is relatively long. In [38] it has been shown for the detection of grapevine fanleaf virus (GFLV) with the magnetic frequency mixing technique that the incubation time (especially at the first step) can be more than halved without compromising the limit of detection. It is also very likely in our case that the assay time could be reduced if needed. In order to facilitate usage in remote areas of the world, it would be advantageous to apply the method presented by Stern et al. [51] to dry the antibody-coated columns for storage at ambient temperature.

## Conclusions

By comparing our detection limit of 0.2 ng/ml and our assay time of a little more than two hours with the above-mentioned literature, we can say that we were able to achieve a sensitive and rapid detection method for CTB. As we used the toxin instead of the bacteria in our research, also illness-creating drinking water can be identified, even if the source organism is not present or has already died, for example, due to disinfection or antibiotic treatment.

To be flexible and to easily compare two different types of MBs, we used a biotin-streptavidin coupling between MB and secondary antibody. A direct chemical coupling between them might be favorable in future assays to reduce the number of assay steps and to achieve a shorter assay time.

We showed that type A beads with 75 nm hydrodynamic diameter yielded a more than tenfold lower detection limit than type B beads with 1010 nm diameter. If magnetic enrichment shall be performed prior to the magnetic assay, bead type A might not be the best choice because they are too small to be efficiently separated with a magnetic gradient field. In that case, the much bigger bead type B might be a better choice because it can be separated well, and might lead to even lower detection limits after performing an efficient magnetic enrichment.

## Supporting information

S1 Table Composition of tap water used in this study. (PDF)

S2 Table Data of ELISA measurements. (XLSX)

S3 Table Measurement data to Figs 3 and 4. (XLSX)

S1 Fig Results of ELISA measurements to find the best antibody combination. (PNG)

S2 Fig Photograph of the magnetic reader in its transport case. Visible is the magnetic reader with touchscreen (lower left corner), the measurement head (upper left corner), power supply (right side), and the cables for power and USB communication to a computer. Additionally, an empty ABICAP column is shown next to the measurement head.
(JPG)

S1 Appendix ELISA experiments for selection of antibody combination. (PDF)

S2 Appendix Inversion of the Hill function. (PDF)

S3 Appendix Additional information on the software. (PDF)
