# Supplementary material for: Sensitive and rapid detection of cholera toxin subunit B using magnetic frequency mixing detection
Source: PLoS One. 2019 Jul 5;14(7):e0219356. doi: 10.1371/journal.pone.0219356 (PMC6611628; doi:10.1371/journal.pone.0219356)
Supplement: S1 Table — (PDF) [file pone.0219356.s001.pdf]

S1 Table. Composition of tap water used in this study

Supplied from Stadtwerke Aachen AG (Aachen, Germany).

Parameter	Unit	Region Aachen 1
**water hardness (calcium carbonate)**	mmol/l	0,5 - 1,5
**acid capacity (K_S 4,3)**	mmol/l	0,5 - 2,0
**pH**	-	8,0 - 9,0
**electrical conductivity**	µS/cm	180 - 320
**calcium (Ca^{2+})**	mg/l	10 - 50
**magnesium (Mg^{2+})**	mg/l	2 - 8
**sodium (Na^{+})**	mg/l	5 - 15
**potassium (K^{+})**	mg/l	1 - 3
**sulphate (SO_4^{2-})**	mg/l	20 - 35
**chloride (Cl^{-})**	mg/l	10 - 25
**nitrate (NO_3^{-})**	mg/l	2 - 10
**nitrite (NO_2^{-})**	mg/l	< 0,1
**ammonium (NH_4^{+})**	mg/l	< 0,04
**fluoride (F^{-})**	mg/l	< 0,2
**cyanide (CN^{-})**	mg/l	< 0,005
**iron (Fe)**	mg/l	< 0,02
**copper (Cu)**	mg/l	< 0,01
**manganese (Mn)**	mg/l	< 0,01
**aluminium (Al)**	mg/l	< 0,05
**lead (Pb)**	mg/l	< 0,002
**cadmium (Cd)**	mg/l	< 0,0002
**nickel (Ni)**	mg/l	< 0,005
**chromium (Cr)**	mg/l	< 0,005
**arsenic (As)**	mg/l	< 0,001
**selenium (Se)**	mg/l	< 0,001
**antimony (Sb)**	mg/l	< 0,001
**mercury (Hg)**	mg/l	< 0,0002
**uranium (U)**	mg/l	< 0,001
