# Supplementary figures and images for: Sensitive and rapid detection of cholera toxin subunit B using magnetic frequency mixing detection
Source: PLoS One. 2019 Jul 5;14(7):e0219356. doi: 10.1371/journal.pone.0219356 (PMC6611628; doi:10.1371/journal.pone.0219356)

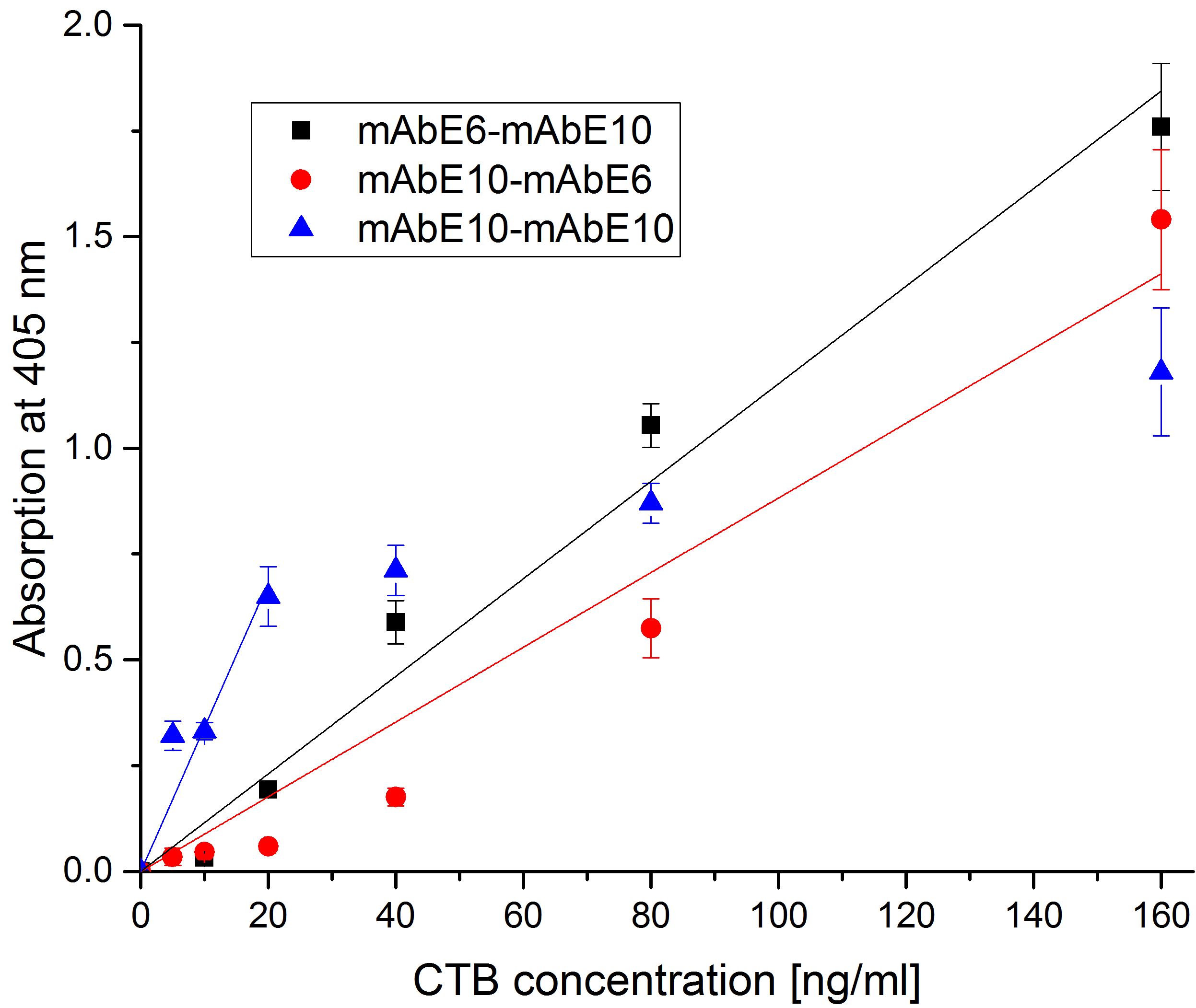

Supplement: S1 Fig — (PNG) [file pone.0219356.s004.png]

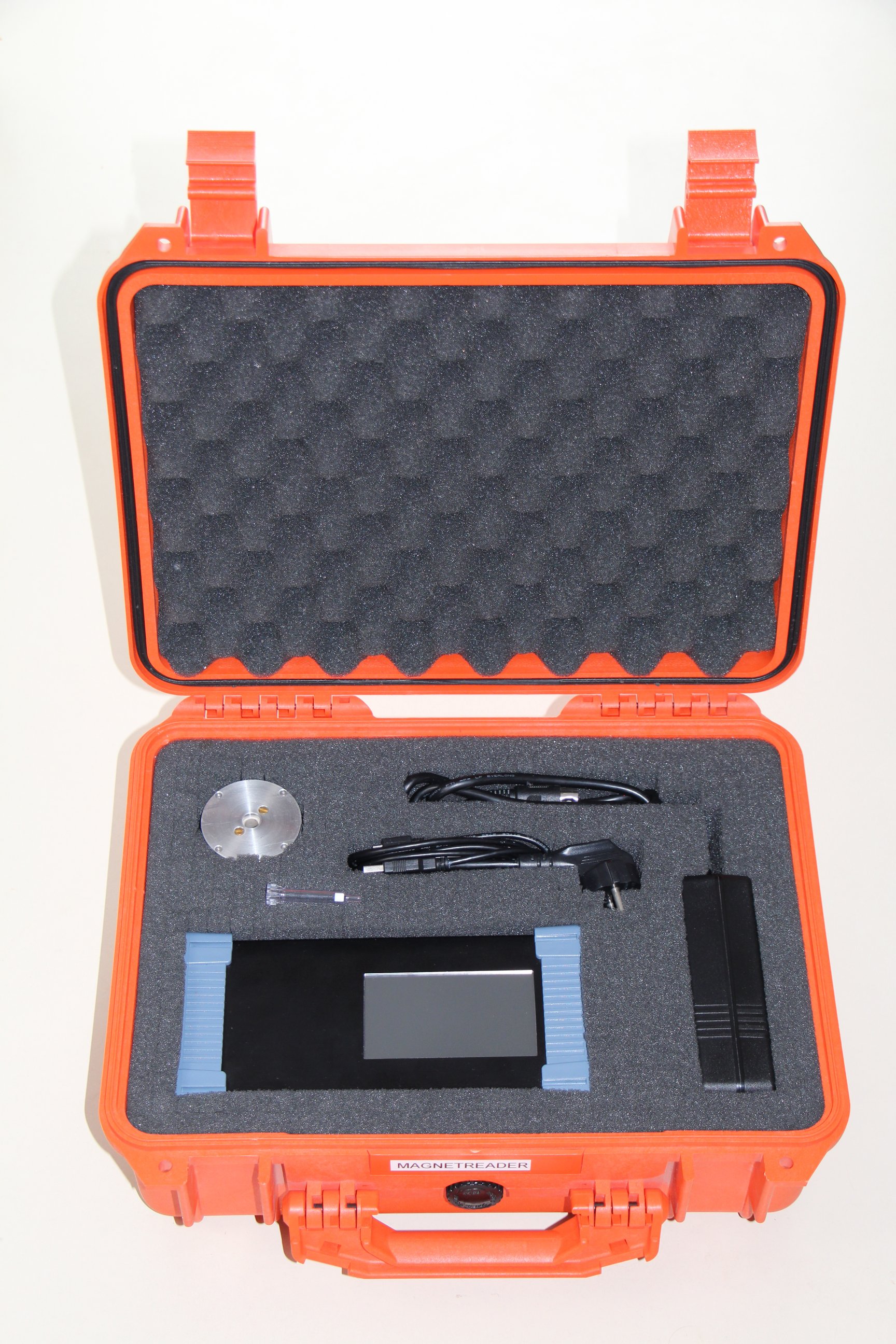

Supplement: S2 Fig — Visible is the magnetic reader with touchscreen (lower left corner), the measurement head (upper left corner), power supply (right side), and the cables for power and USB communication to a computer. Additionally, an empty ABICAP column is shown next to the measurement head. (JPG) [file pone.0219356.s005.JPG]
