# Supplementary material for: Sensitive and rapid detection of cholera toxin subunit B using magnetic frequency mixing detection
Source: PLoS One. 2019 Jul 5;14(7):e0219356. doi: 10.1371/journal.pone.0219356 (PMC6611628; doi:10.1371/journal.pone.0219356)
Supplement: S1 Appendix — (PDF) [file pone.0219356.s006.pdf]

S1 Appendix. ELISA experiments for selection of antibody combination

To select the antibodies used in the immunomagnetic sandwich assay, we performed standard ELISAs and measured the absorption at 405 nm. There we tested the two antibodies in different combinations. Each experiment was performed three times and the mean and standard deviation were used for further analysis. From these experiments, we chose the best combination and transferred it to the immunomagnetic assay.

The results are shown in S1 Fig and the values can be found in S2 Table.

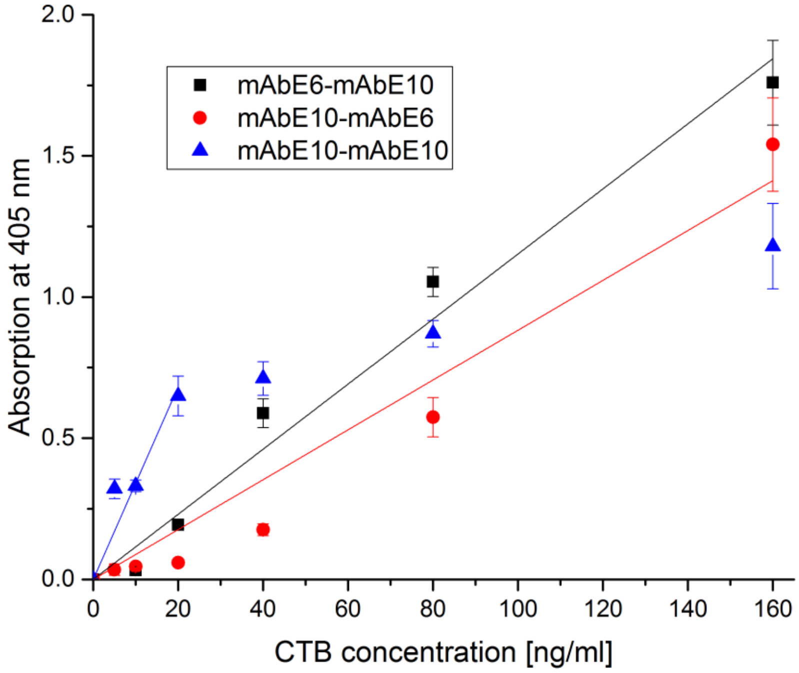

S1 Fig. Results of ELISA measurements to find the best antibody combination. The naming scheme is always coating hyphen secondary antibody. Shown are the mean and standard deviation. Additionally linear fits are applied to the suitable ranges.

It can be seen that the ELISA measurements of mAb E10 as coating and secondary antibody goes into saturation very fast and leaves the linear region. Because of this only up to 20 ng/ml a linear fit can be performed well. The other combinations using both different antibodies do not show this behavior in this range. Additionally by using two different antibodies which bind to different epitopes it would be possible to even detect monomeric CTB. The combination of mAb E6 as coating antibody and mAb E10 results in the highest measurement signals and lowest detection limits.
