# Supplementary material for: Sensitive and rapid detection of cholera toxin subunit B using magnetic frequency mixing detection
Source: PLoS One. 2019 Jul 5;14(7):e0219356. doi: 10.1371/journal.pone.0219356 (PMC6611628; doi:10.1371/journal.pone.0219356)
Supplement: S2 Appendix — (PDF) [file pone.0219356.s007.pdf]

S2 Appendix. Inversion of the Hill function

Stepwise inversion of the Hill function

$$y = end \cdot \frac{x^n}{k^n + x^n} \quad \text{(A1)}$$

$$y \cdot (k^n + x^n) = end \cdot x^n \quad \text{(A2)}$$

$$y \cdot k^n = (end - y) \cdot x^n \quad \text{(A3)}$$

$$x^n = \frac{y \cdot k^n}{end - y} \quad \text{(A4)}$$

$$x = k \cdot \left(\frac{y}{end - y}\right)^{\frac{1}{n}} \quad \text{(A5)}$$

This formula can be used as long as the found fit parameter for *end* is larger than the *y* value of interest.
