# Supplementary material for: Sensitive and rapid detection of cholera toxin subunit B using magnetic frequency mixing detection
Source: PLoS One. 2019 Jul 5;14(7):e0219356. doi: 10.1371/journal.pone.0219356 (PMC6611628; doi:10.1371/journal.pone.0219356)
Supplement: S3 Appendix — (PDF) [file pone.0219356.s008.pdf]

S3 Appendix. Additional information on the software

The software used for the analysis of the measurement results in this work is a home-written python solution. It uses the curve fitting function of the SciPy package for python. To find a fit function, the measured values with their standard deviations (if available) and the corresponding concentrations are loaded into the software from a text file or manually entered using a mask which appears after pressing the “Create FitData File” Button (see S3 Fig). The user can decide if the standard deviation should be taken into account during the fitting or not. Different measurement files can be loaded at the same time and fitted in one run. After the fitting the measured calibration points (blue) together with their standard deviation (blue) as well as the found fit function (black) is displayed. The formula of the fit function together with its parameters is also shown. Additionally the software directly calculates the detection limit, as described above, and displays it as a vertical line (red), and in text form. The displayed image as well as the fit parameters can be directly saved for later analysis or be used to determine the concentration of unknown samples. We compared the results of our software with the ones found using the established software Origin 2015 (OriginLab Corporation, Northampton, United States of America). Our software yielded almost the same fitting parameters and R^2 values as Origin did.

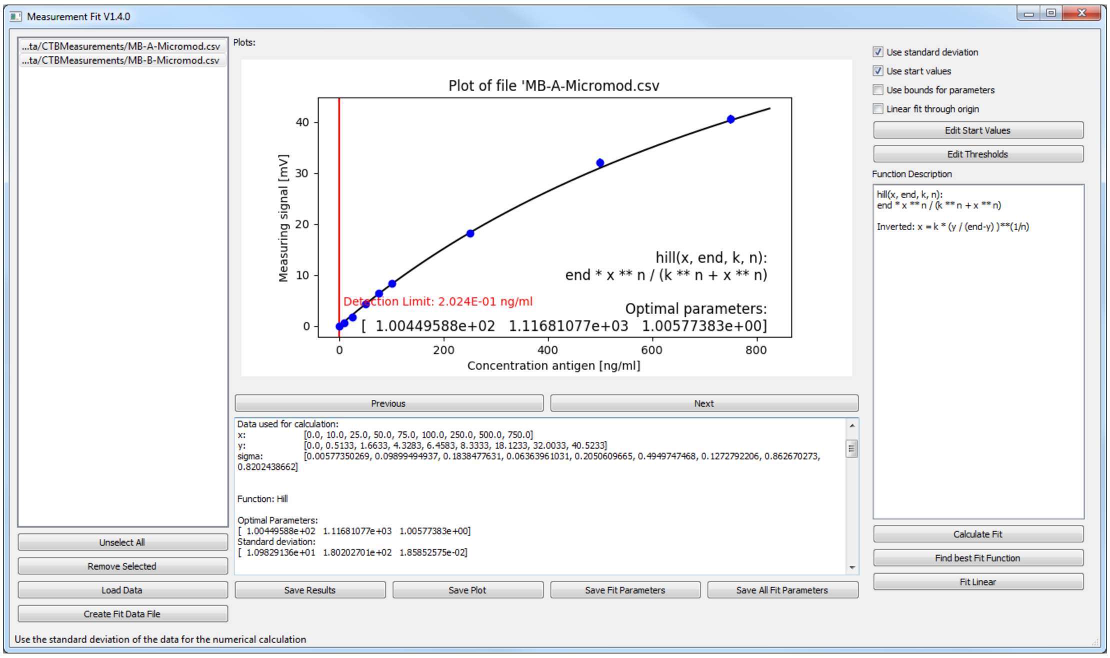

S3 Fig. Screenshot of the software to calculate the fit parameters of the Hill function for a given dataset. Here, two datasets are loaded and fitted by taking the standard deviation of the points

into account. The graphical representation of the calibration measurement for CTB using the magnetic bead A is displayed. The detection limit was found to be about 0.2 ng/ml (indicated in red).

We wrote a second software for calculating the concentration of a sample by entering its measured value in mV. This program loads the previously generated calibration model with its parameters, and displays the calibration points, parameters and calculated detection limit (see Fig 3 in the paper). When the user enters a measured value, the software indicates this value on the fit curve. If the corresponding standard deviation is given, it also indicates to which range of concentrations it is linked to. By plotting the function and the point together with its standard deviation, it becomes obvious why the same absolute standard deviation has a different outcome in the concentration interval at different measured signals, due to the nonlinear form of the curve and its saturation behavior.
